# The challenges of the COVID‐19 pandemic: Approaches for the elderly and those with Alzheimer's disease

**DOI:** 10.1002/mco2.4

**Published:** 2020-05-15

**Authors:** Qing Zhang, Weihong Song

**Affiliations:** ^1^ Townsend Family Laboratories Department of Psychiatry The University of British Columbia Vancouver British Columbia Canada

**Keywords:** Alzheimer's disease, coronavirus, COVID‐19, economic, healthcare, information access, mental health, SARS‐CoV‐2, the elderly

## Abstract

Coronavirus disease 2019 (COVID‐19) is an infectious disease that since its outbreak in December 2019 has become a global pandemic. COVID‐19 is caused by the previously unknown coronavirus SARS‐CoV‐2. The elderly are the most vulnerable to COVID‐19, and have the highest mortality of the afflicted. Similar patterns have been observed in epidemics and pandemics throughout the 20th and the beginning of the 21st centuries. In this article, we review some unique challenges the elderly and people with Alzheimer's disease face during the COVID‐19 pandemic and suggest approaches that could be taken from healthcare and social approaches to better handle this pandemic.

**Abbreviations**
COVID‐19: coronavirus disease 2019
SARS: severe acute respiratory syndrome

## 1 INTRODUCTION

Coronavirus disease 2019 (COVID‐19) is an ongoing pandemic caused by the previously unknown coronavirus SARS‐CoV‐2.^1^ A cluster of pneumonia cases were reported in December 2019, and it has since been gradually contained by effective management strategies in China and a few countries.^2^ The COVID‐19 has become a worldwide pandemic.

As of 2:00 am CEST on April 20*, there have been 2 319 066 confirmed cases and 157 970 deaths worldwide, bringing the global case‐fatality rate (CFR) to 6.81%, with 4.44% in Canada, 4.72% in the United States, 5.51% in China, 6.24% in Iran, and 13.22% in Italy.^3^

The elderly have been hit hardest with the highest rate of incidence, the most severe symptoms, and the highest death rate. The CFR reached as high as 8% in patients aged 70‐79 years old, and 14.8% in patients older than 80 years of the reported 72 314 cases in China.^4^ Italy has the second highest number of cases worldwide. The mean age of 355 deaths from COVID‐19 in Italy was 79.5 years old,^5^ and people aged 70 and older accounted for 87.9% of the 1625 deaths as of 15 March.^6^ Ageing is the greatest contributing factor towards risk of developing COVID‐19. Therefore, protection of the vulnerable elderly populations, particularly those with dementia, from COVID‐19 and managing the long‐term effects for those infected is a global issue that should not be neglected and requires urgent attention.

It has been demonstrated throughout epidemiological and historical records that elderly populations are particularly vulnerable during epidemics and pandemics. One pathogen that has particularly threatened the aged population is the influenza virus. The 1918 influenza pandemic, also known as the Spanish flu, emerged nearly simultaneously around the world in 1918‐1919. It resulted in one of the most disastrous pandemics in recorded history: the disease had become symptomatic in approximately one third of the population worldwide,^7^ leading to an extraordinary global death toll that was estimated to be from 50 to 100 million.^8^ Influenza typically has higher rates of mortality in infants and seniors, shown by a “U‐shaped” age‐specific mortality. The Spanish flu exhibited a “W‐shaped” age‐specific influenza mortality, with a third peak in young adults.^7^ The 1918 influenza was caused by the H1N1 influenza A virus. Since then, all subsequent influenza A pandemics have been caused by descendants of the 1918 influenza virus^9^: The 1957 “Asian influenza” caused by H2N2, resulted in 1‐2 million deaths; The 1968 “Hong Kong influenza” caused by H3N2, led to 700 000 deaths; The 2009 “Swine influenza” caused by H1N1, ended with 363 000 deaths.^7^ By analyzing the age distribution of influenza‐related deaths in the United States during the 20th century, Simonsen et al found that compared to individuals below 65, those above 65 had an increasing risk ratio across the three pandemics in the 20th century, rising from 0.3:1 in the Spanish flu to 18:1 in 1957 H2N2 pandemic, and 13:1 in 1968 H3N2 pandemic. The risk ratio of the individuals above 65 fluctuated during the annual H3N2 epidemics from 1968 to 1995, reaching 281:1 in 1987, and with excess mortality exclusively in the aged group in 1982.^10^

At the beginning of the 21st century, novel coronaviruses became a major threat to public health worldwide, especially for the aged population. The severe acute respiratory syndrome (SARS) from November 2002 to July 2003, caused by a novel coronavirus (SARS‐CoV), was the first epidemic of the 21st century. It had infected 8096 people and resulted in 774 deaths across 26 countries worldwide, with a CFR at 9.6%.^11^ Age is an independent risk factor (Relative risk = 1.8) for death or intensive care unit (ICU) admission, and the risk for development of acute respiratory distress syndrome was increased by 28‐fold (95% CI, 3.1‐253.3) in the population aged 61‐80 years old.^12^ The Middle East respiratory syndrome (MERS), first reported in Saudi Arabia in 2012, is a pandemic‐prone ongoing disease caused by the novel coronavirus MERS‐CoV.^13^ By the end of January 2020, MERS has affected 2519 patients, resulting in 866 deaths, with a high CFR at 34.3%. The elderly continue to be the most vulnerable to infection and death.^14^

COVID‐19 was presumed to be more threatening to elderly persons since it was first reported, with the mean age of patients being 49‐55.5 years.^15^, ^16^ In a recent study analyzing the clinical characteristics of 1099 laboratory test‐confirmed COVID‐19 patients throughout China (11 December to 29 January), this threat to the aged population was highlighted again.^17^ Although the median age for all 1099 patients was 47, the median ages for nonsevere and severe cases were 45 and 52, respectively. Furthermore, the age distribution revealed more aged patients in the severe group: patients aged 65 and older constituted 27% of the severe cases, whereas the same age group only accounted for 12.9% of the nonsevere patients. Additionally, the incidence of coexisting disorders was 38.7% in the severe group, compared to 21% in the nonsevere group. Many of these coexisting diseases were more prevalent in older populations: hypertension (severe vs nonsevere: 23.7% vs 13.4%), diabetes (16.2% vs 5.7%), coronary heart disease (5.8% vs 1.8%), chronic obstructive pulmonary disease (3.5% vs 0.6%), and cerebrovascular disease (2.3% vs 1.2%).^17^ These coexisting conditions can render older individuals more vulnerable to COVID‐19, and exacerbate the severity of the disease for elders. In another retrospective study investigating the clinical courses of 52 critically ill patients in Wuhan, China (24 December to 26 January), the threat to the aged population was once more indicated to be severe among nonsurvivors.^18^ The average age of all 52 critically ill patients was 59.7, with the mean age of 20 survivors and 32 nonsurvivors being 51.9 and 64.6, respectively. The age distribution of nonsurvivors also showed an ageing trend, in which people aged ≥60 made up 62% of the nonsurvivors, compared to 35% of the survivors. The nonsurvivors also had higher overall incidence for coexisting disorders (nonsurvivors vs survivors: 50% vs 25%), with higher prevalence of common diseases in elders such as cerebrovascular disease (22%) and diabetes (22%).^18^ Another retrospective study of 138 cases from 1 January to 28 January reached the similar conclusion that patients treated in the ICU were older (66 vs 51 years), and had higher incidence of comorbidities (72.2% vs 37.3%).^19^ A retrospective study of 82 death cases revealed that 80.5% died patients were older than 65, with the median age being 72.5, and that 76.8% cases had comorbidities.^20^ Reports from Italy showed that 85.5% of patients presented two or more pre‐existing disorders.^21^

Facing the growing threat of COVID‐19 and drawing upon the lessons learnt before, World Health Organization (WHO) has called upon the whole world to take rapid and concrete actions to stop the pandemic. Guidance on how to protect the most vulnerable group, the elderly population and particularly those with Alzheimer's disease (AD), becomes urgent and paramount.

China's recent COVID‐19 containment strategy has now become effective in containing their outbreak, as shown by the precipitous drop in new confirmed cases. Using a stochastic transmission model, it was predicted that efforts should be made in tracing and isolating 80% of symptomatic contacts to control 80% of outbreaks. It has been acknowledged that effective case isolation and contact tracing can control a new outbreak of COVID‐19.^22^ The isolation strategy in China indeed has significantly contributed to the virus containment. There were concerns about whether the isolation approach by China can be carried out in other countries due to the differences in culture and political systems.^2^ Shortly after the debate, Italy first imposed a national lockdown on 9 March 2020, followed by more countries including Spain, Germany, India, France, and Canada with the hope to flatten the curve. Although some methods in China are not applicable to other countries, certain strategies can be viable in protecting the most vulnerable ageing population. Based on the early information on the COVID‐19 outbreak in China, the Center for Disease Control and Prevention (CDC) in the United States published a prevention guideline for people at higher risk, including older adults and people with chronic medical conditions, especially heart disease, diabetes, and lung diseases.^23^ The guideline underscores personal hygiene, social distancing, preparation of supplies (medical and household), and community support,^23^ which are practical and effective approaches as demonstrated by the proper containment in China.

The world is facing the public health challenge of ageing populations in the 21st century. The ever increasing aged population has not only resulted in an increase in physical and mental morbidities but also shown substantial implications from economic, societal, and public health perspectives.^24^ Here, we reviewed some unique aspects of aged populations and the seniors with AD that may render them susceptible to COVID‐19, and the corresponding strategies to cope with the threat to ageing populations.

## 2 LIVING ARRANGEMENT AND COMMUNITY SUPPORT

The increase in ageing populations has been coupled with a sharp growth of elderly people living alone over the past century. As of 2013, approximately 27 million people aged 65 and older lived alone across the 28 states of the European Union and 12 million in the United States.^25^ The elderly living independently have some unique challenges, especially health‐related issues.^25^ Aged people living alone may lack health‐associated support from families, such as care during illness, surveillance, and household labor. This living arrangement adds significant challenges in coping with the COVID‐19 pandemic. Communities may wish to establish a platform to monitor their daily activities and assist them in the ordering and delivery of necessities.

## 3 INFORMATION ACCESS AND PUBLIC EDUCATION

Compared to younger generations, elders have very limited access to the Internet, the most important source of information in today's world. As a result, their ability to receive news and updates in a timely manner is reduced. Public officials and the press should work to disseminate only accurate scientific information regarding the COVID‐19 pandemic in a timely and truthful manner to ease people's minds, with the aim to prevent panic and hysteria. Although real‐time release of news and information may result in panic buying, this is a preferable alternative to being forced to deal with a pandemic once it has spiraled out of control. Information disseminated by authority figures and news outlets (which are then often filtered through social media) is the main source by which the public receives information, which thus influences the behavior of the population in these times of crisis. Although the Internet and social media can often result in misinterpretations by the general public, as it pertains to older populations (who are less likely to receive news from these sources), greater attention should be paid to traditional media to which these elders would be likely to have access, such as television, newspaper, and radio.

## 4 HEALTHCARE OF AGE‐RELATED DISEASES

Ageing has been a major risk factor for chronic diseases, including cardiovascular diseases, diabetes, cancer, and neurodegenerative diseases.^26^ Many of these common morbidities render aged people more susceptible to infections and adverse outcomes, with COVID‐19 being the newest threat. The healthcare system can put aged people at risk of infection unintentionally, given the complex pathogenic environment in the healthcare setting for the elderly. Therefore, healthcare system and aged individuals should try to minimize the chance of unessential doctor visits, such as annual physical examination and dental cleaning. As more countries have joined the list of national lockdown, there is expected to be decreased amounts of outpatient visit for diseases other than COVID‐19 in hospitals, and the elders are facing increased difficulties in getting access to regular healthcare. Healthcare staffs should pay more attention to, and may set aside dedicated efforts to monitor aged individuals with underlying diseases that require regular monitor such as hypertension. These aged patients are encouraged to contact their doctors by phone or online, and doctors can prescribe some back‐up medicines for long term, which may be delivered to patients, to avoid unnecessary exposure.

## 5 MENTAL HEALTH ISSUE DURING THE PANDEMIC

The outbreak of COVID‐19 and high rate of mortality will inevitably cause mental health problems. Seniors living alone are more prone to have innate lonely and helpless feelings, and these existing mental disorders could be exacerbated during COVID‐19 pandemic.^27^ Therefore, greater attention should be paid to their mental wellbeing. Compared to younger generations, due to limited access to Internet for news and updates in a timely manner, seniors may not be well informed or prepared for the pandemic. More efforts should be made in communicating new knowledge of COVID‐19 to these populations. Psychological counselling, psychiatric care, and treatment play a critical role to prevent panic during pandemic and in dealing with further suffering from post‐pandemic traumatic stress disorder. During national lockdown, however, there are even less options that elders could have for their mental care. Under the current situation, psychiatric clinics can monitor their aged patients who need regular outpatient visits by phone and prescribe drug through delivery. For the mental health of elders without pre‐existing mental disorders, psychological services may collaborate with local communities that assist the daily life of the elderly, to monitor their mental states and provide psychological first aid counselling and care in real‐time.

## 6 NURSING AND ALZHEIMER PATIENTS

Although elderly people in nursing homes typically have limited opportunities to connect with the outside world, asymptomatic healthcare staffs could transmit the virus unknowingly and unintentionally, which has been the case in several cities around the world. Once a virus carrier gets into the relatively isolated location with a number of aged individuals with limited mobility, there could be a sudden outbreak that is hard to control. Inside nursing homes and assisted care facilities, a large group of elders that need special attention during this COVID‐19 pandemic are AD patients. Pneumonia is the leading cause to death in the elderly with dementia.^28^, ^29^ An 18‐month perspective study of 323 residents with dementia in 22 nursing homes showed that 41.1% of the residents with dementia had one or more pneumonia episodes, and pneumonia was responsible for a 46.7% 6‐month mortality rate.^28^ An autopsy study of 218 subjects with dementia showed that pneumonia was the leading cause to death in 66.3% cases, followed by cardiovascular disease (16.3%).^29^ The cognitive impairment and memory loss endemic make the elderly with AD more difficult to prevent and cope with the infection. Protecting the seniors, particularly those with AD, becomes urgent and paramount. Therefore, staffs working in nursing home and other assisted care facilities should take every precaution to prevent the spread of COVID‐19 to not only themselves, but also to the vulnerable aged population. All caregivers should be tested for SARS‐CoV‐2 virus and to have all the seniors to self‐isolate for a period of time and outside visiting to the patients should be limited. Although this strategy could be very effective in preventing and containing COVID‐19, extra cares and supports for the seniors with AD should be considered with this approach to prevent virus transmission and to treat COVID‐19 if being developed.

Apart from protecting those currently suffering from AD, who are at greater danger of COVID‐19 infection, another important question is that whether the pandemic could have a potential impact on brain health and dementia pathogenesis. AD is the most common neurodegenerative disorder leading to dementia, and there is a growing body of evidence that has implicated the infection in the pathogenesis of AD. Herpes viruses have been implicated in AD development by animal and human studies. Whether the infection is a risk factor for AD development is not clear.^30^ H5N1 influenza virus was shown to have the potential to travel to the central nervous system of C57BL/6J mice, resulting in neuroinflammation, neuronal loss, and protein aggregation.^31^ Evidences regarding the association between influenza pandemics in the 20th century and AD development, however, are sparse. The majority of AD cases are late‐onset without a known cause. Ageing is the most significant risk factor for AD, but additional environmental risk factors contribute to the development of dementia.^32^ Whether coronaviruses including SARS‐CoV, MERS‐CoV, and recent SARS‐CoV‐2 play any role in developing AD remains an issue to be addressed. Future studies are warranted to investigate the association between infection and AD pathogenesis, so as to develop effective means to break the potential vicious loop of infection‐AD‐pneumonia‐associated death.

In summary, the elderly as a population face the greatest challenges posed by COVID‐19 from physical, mental, and social perspectives, and their affliction will inevitably lead to a huge burden being placed upon healthcare systems and containment. The WHO has “called every day for countries to take urgent and aggressive action,”^33^ and here we advocate that governments and healthcare systems worldwide should pay more attention to the most vulnerable elders, particularly those with AD, helping them and the whole world overcome the pandemic.

## AUTHOR CONTRIBUTIONS

WS conceived the study. QZ and WS analyzed the data and wrote the paper. All authors reviewed the manuscript.

## CONFLICT OF INTEREST

The authors declare no conflict of interest.

## DATA AVAILABILITY

The data that support the findings of this study are available from the corresponding author upon request.

## Footnotes

* The data was updated after the paper was accepted.
